# Growth model for arbuscular mycorrhizal fungi

**DOI:** 10.1098/rsif.2007.1250

**Published:** 2007-12-11

**Authors:** A Schnepf, T Roose, P Schweiger

**Affiliations:** 1 Department of Forest and Soil Sciences, Institute of Soil Science, BOKU-University of Natural Resources and Applied Life SciencesPeter Jordan-Straße 82, 1190 Vienna, Austria; 2 Oxford Centre for Industrial and Applied Mathematics and Centre for Mathematical Biology, Mathematical Institute, University of Oxford24–29 St Giles, Oxford OX1 3LB, UK

**Keywords:** arbuscular mycorrhizal fungi, extraradical mycelium, growth model, hyphal length density, hyphal tip density

## Abstract

In order to quantify the contribution of arbuscular mycorrhizal (AM) fungi to plant phosphorus nutrition, the development and extent of the external fungal mycelium and its nutrient uptake capacity are of particular importance. We develop and analyse a model of the growth of AM fungi associated with plant roots, suitable for describing mechanistically the effects of the fungi on solute uptake by plants. The model describes the development and distribution of the fungal mycelium in soil in terms of the creation and death of hyphae, tip–tip and tip–hypha anastomosis, and the nature of the root–fungus interface. It is calibrated and corroborated using published experimental data for hyphal length densities at different distances away from root surfaces. A good agreement between measured and simulated values was found for three fungal species with different morphologies: *Scutellospora calospora* (Nicol. & Gerd.) Walker & Sanders; *Glomus* sp.; and *Acaulospora laevis* Gerdemann & Trappe associated with *Trifolium subterraneum* L. The model and findings are expected to contribute to the quantification of the role of AM fungi in plant mineral nutrition and the interpretation of different foraging strategies among fungal species.

## 1. Introduction

Arbuscular mycorrhizae are mutualistic symbiotic associations between plant roots and specific soil fungi and are formed by more than 90% of all terrestrial plants (Brundrett 2002). They may offer several benefits to the host plant, including improved mineral nutrition, especially phosphate, and enhanced growth (Smith & Read 1996). The fungi are obligate symbionts and benefit from the symbiosis by receiving photosynthates from the plant. Arbuscular mycorrhizal (AM) fungi grow inside plant roots as well as in the surrounding soil. The root-internal mycelium grows mainly intracellularly, but enters root cortex cells to form highly branched structures called arbuscules, where the transfer of, for example, phosphorus from fungus to root is thought to occur. The root-external mycelium spreads several centimetres away from root surfaces into the soil. It can thereby access poorly mobile soil nutrients from outside the nutrient depletion zone formed around actively absorbing roots. Many metres of fungal hyphae may be produced per gram of soil (Miller *et al.* 1995). This results in a greatly increased surface area for plant nutrient absorption. A diagram of an arbuscular mycorrhiza is shown in figure 1.

In spite of the worldwide occurrence of the AM symbiosis, mathematical models on mycorrhizae are scarce. Neuhauser & Fargione (2004) presented an ecologically based model of mutualism–parasitism with specific application to plant–mycorrhiza interaction. This model is based on the classical host–parasite Lotka–Volterra model and hence is not spatially explicit.

In order to quantify the increased surface area provided by the fungal mycelium and its effect on nutrient uptake, it is necessary to quantify the dynamics of the external fungal mycelium. A first model to quantify the contribution of the external fungal mycelium to plant phosphorus uptake was developed by Schnepf & Roose (2006). Their predicted root inflow was low compared with published data (Tinker & Nye 2000; McGonigle & Fitter 1988). However, the result of Schnepf & Roose (2006) was derived on the scale of a single mycorrhizal root, while the values of Tinker & Nye (2000) and McGonigle & Fitter (1988) were derived on a whole-plant basis, where a lot of additional processes such as root growth and root colonization have to be considered. Schnepf & Roose (2006) also analysed phosphorus translocation within the mycelial network and showed that it is so fast that the nutrient availability cannot be the rate-limiting step for the mycorrhizal pathway of phosphorus into the root.

Models for growth of non-mycorrhizal mycelial fungi are directed at cell physics (Bartnicki-Garcia *et al.* 2000), physiological processes and population dynamics (Edelstein 1982; Boswell *et al.* 2003) or biochemical processes (Tlalka *et al.* 2003). Edelstein (1982) describes colony growth based on average properties of single hyphae. The model includes processes such as elongation of fungal hyphae, creation of new branches, fusion of a tip with another tip or its neighbouring hypha (anastomosis) and hyphal death. This model has been extended by Stacey *et al.* (2001) to model the transmission of soil-borne fungal pathogens by colony growth from infectious to susceptible plants. Boswell *et al.* (2003) extended the work of Edelstein (1982; Edelstein & Segal 1983) to include physiologically derived uptake and translocation mechanisms of a growth-limiting substrate within the fungal colony. An approach rather different from such continuous models is presented by Meškauskas *et al.* (2004). The vector-based ‘neighbour-sensing model of hyphal growth’ is directed at simulating mycelial morphology explicitly and in three dimensions using movement rules similar to cellular automata. This model approach is illustrative, but it appears difficult to assess the physiological context and compare it quantitatively with experiments. This difficulty was overcome by a hybrid cellular automata model recently derived by Boswell *et al.* (2007) and Davidson (2007*b*), where a discrete model describes the fungal mycelium while substrate concentrations are modelled as continuous variables. The parameters in this model were derived from experimental observations. Falconer *et al.* (2005, 2007) present a physiologically based model for mycelium growth that incorporates transport and recycling of biomass. They show how hyphal-scale features and transport are involved in the production of colony-scale features. Impact of colony-scale context on local behaviour within the mycelium is explained in terms of the dynamics of resource uptake, biomass remobilization and movement. However, this model is based on a qualitative flux of colony biomass and does not incorporate hyphal-scale mechanisms such as branching. For reviews on fungal growth modelling, see Prosser (1993), Bezzi & Ciliberto (2004) and Davidson (2007*a*).

In this paper, we aim to develop and analyse a model for growth of AM fungi, which can be used to create a sink term for plant solute uptake models and represents all necessary processes for mycorrhizal growth. Such a model can be validated against experiments that measure the contribution of the AM fungi to plant phosphorus uptake. For this final goal, a suitable representation of mycorrhizal colony growth is needed. The study of mycelial fungi involves spatial scales ranging from micrometres or less to centimetres, or in some cases even kilometres (Davidson 2007*a*), and it is our first task to choose the spatial scale most appropriate for our purpose. To develop a sink term for mycorrhizal root uptake models, we need to know the local surface area of the hyphal network at given distances away from the root surface, i.e. a representation on the colony scale. The Edelstein model describes the dynamics of mycelium formation based on microscopic processes such as branching and anastomosis. It has been shown that these processes are also relevant for mycorrhizal fungi (Giovanetti *et al.* 2001). Therefore, it seems to be a promising starting point for the development of a model for growth of mycorrhizal fungi. However, it is necessary to define a model boundary condition that represents the interface between root and fungus, which is characteristic of the mycorrhizal symbiosis.

We begin by deriving a model for growth of AM fungi. We then calibrate it against the experimental data of Jakobsen *et al.* (1992) and provide an analysis and discussion of model results. Model calibration results will be useful to find out differences in different species of mycorrhizal fungi and their behaviour, with particular emphasis on branching and anastomosis.

## 2. Model formulation

We describe the mycelium by two continuous variables: the hyphal length density *ρ* and the hyphal tip density *n*. The hyphal length density is the variable that is most easily compared with experimental data. Hyphal tips are important, because growth is thought to occur due to the elongation of the region just behind the tips (Edelstein 1982; Gow & Gadd 1994; Bartnicki-Garcia *et al.* 2000). Therefore, we can describe the movement of hyphal tips, with the tips leaving a ‘trail’ of newly created hyphae behind them. Conservation of tips gives the following equation for the hyphal tip density:

(2.1) $$\frac{\partial n}{\partial t}=-\nabla \cdot (nv)+f,$$

where ***v*** is the rate of tip movement and *f* is the rate of creation or destruction or both of hyphal tips, i.e. branching, death and anastomosis. Mycorrhizal fungi are known to branch mainly apically where one tip splits into two. In the simplest case, branching and tip death are linearly proportional to the existing tip density, i.e. $f=b_{n}n-d_{b}n$, where *b*_*n*_ is the tip branching rate and *d*_*n*_ is the tip death rate. Alternatively, if we assume that branching decreases with increasing tip density and ceases at a given maximal tip density, the term *f* becomes $f=b_{n}n(1-(n/n_{\text{max}}))-d_{n}n$. From a biological point of view, this behaviour could be due to the competition between hyphal tips for space and growth resources when the tip density is large.

The fusion of two hyphal tips or a tip with a hypha can create interconnected networks by means of anastomosis. The functional form of *f* describing tip–hypha and tip–tip anastomosis is $f=-a_{2}n\rho -a_{1}n^{2}$ (Edelstein 1982), where *ρ* is the hyphal length density and *a*_1_ and *a*_2_ are the tip–tip and tip–hypha anastomosis rate constants, respectively.

Considering nonlinear branching, tip death and tip–hypha and tip–tip anastomosis, the most general functional form of *f* is $f=b_{n}n(1-(n/n_{\text{max}}))-d_{n}n-a_{2}n\rho -a_{1}n^{2}$. However, if required, it could include more terms or a more detailed description of the existing terms where the constants are replaced by functions that could be dependent on parameters related to the fungal physiology and/or its interaction with the environment. Mycelial fungi are capable of translocating nutrients within the hyphal network, e.g. from places of high nutrient concentrations to places where nutrients are scarce (Bezzi & Ciliberto 2004). Recent models of non-mycorrhizal mycelial fungi assume that growth is dependent on the internal and external concentrations of the growth-limiting nutrient carbon (Boswell *et al.* 2003; Davidson 2007*a*). AM fungi receive carbon exclusively from their host plant, and it would be necessary to model this aspect of the mycorrhizal symbiosis by including the transfer of carbon from the host plant to the fungus and translocation within the mycelium to the fungal tips where growth occurs. However, owing to the lack of experimental data, we are unable to quantitatively include this in the current model. At this stage, we focus on the effect of the increased surface area provided by the external fungal mycelium on plant availability of nutrients such as phosphate. External and internal phosphate concentrations were considered by Schnepf & Roose (2006), where they considered nutrient uptake and translocation within the fungal network towards the plant root. They showed that translocation was so fast that it cannot be a limiting step in plant phosphate supply or fungal growth.

The hyphal length density is dependent on tip density and movement and hyphal death. The increase in hyphal density can be written as *n*|***v***|, i.e. the length of the trail left behind as tips move through the unit volume. We consider hyphal death to be linearly proportional to the hyphal length density *ρ*, so that the equation describing the hyphal length density is

(2.2) $$\frac{\partial \rho }{\partial t}=n|v|-d\rho ,$$

where *d* is the rate of hyphal death.

To close the growth model described by equations (2.1) and (2.2), we need to apply initial and boundary conditions. For AM fungi, we have to consider the boundary interface between root and fungus because the fungus cannot grow without the root. In future, it would be interesting to couple the model for external fungal mycelium with a model of root colonization, which would also define the root–fungus interface. However, in a first approach, we define a boundary condition where the hyphal tip density is prescribed by a given function that may depend on other variables such as *t*, *ρ* or *n*. Its shape could be determined from experimental data. Owing to the lack of data that would enable us to develop such a function, we use a simple linear function

(2.3) $$n(r_{0},t)=at+n_{0,b},$$

where *n*_0,*b*_ is the tip density initially present at the boundary and *a* is a boundary proliferation parameter. If the soil surrounding the root is initially fungus free, we have as initial condition that *ρ*=*n*=0.

Considering the growth of fungal hyphae from a cylindrical root in radial polar coordinates, the model equations and initial and boundary conditions are

(2.4) $$\frac{\partial n}{\partial t}=-\frac{1}{r}\frac{\partial (rnv)}{\partial r}+f,\;t\geq 0,\;r\geq r_{0},$$

(2.5) $$\frac{\partial \rho }{\partial t}=nv-d\rho ,\;t\geq 0,\;r\geq r_{0},$$

(2.6) $$n=0,\;t=0,\;r\geq r_{0},$$

(2.7) $$\rho =0,\;t=0,\;r\geq r_{0},$$

(2.8) $$n=at+n_{0,b},\;r=r_{0},\;t>0,$$

where *r*_0_ is the root radius and *v* is the speed of radial tip movement.

### 2.1 Model analysis and solutions

For analysis and solution of the model described by equations (2.4)–(2.8), we apply the technique of non-dimensionalization (Fowler 1997), a process of changing variables by scaling so that the new variables have no units. This procedure leads to a simpler form of equations with fewer parameters that are all dimensionless. These parameters describe the relative importance of different processes included in the model compared with each other and determine the behaviour of the solution. However, the same qualitative behaviour may be the result of different combinations of the dimensional parameter values. Thus, non-dimensionalization tells us about the behaviour of the system for a wide range of dimensional parameter values.

For the simplest form of the function *f*, which considers linear branching, no anastomosis and a constant flux of tips across the root–fungus interface, *f* is given by

(2.9) f=bn,

where $b=b_{n}-d_{n}$ is the net rate of creation of hyphal tips. Henceforth, we call this model the linear branching model. Its solution can be found analytically using the method of characteristics (Ockendon *et al.* 2003). The full derivation is given in appendix A. We compare the hyphal length density with experimental data in §4. The solution for the hyphal length density is given by

(2.10) $$\rho (r,t)=\frac{1}{d}\frac{r_{0}}{r}\text{exp}\left(\frac{b}{v}(r-r_{0})\right)\{\begin{array}{ll} ab\left(tv-r+r_{0}-\frac{v}{d}\right)+vn_{0,b}+\left(a\frac{vb}{d}-vn_{0,b}\right)\text{exp}\left(\frac{d}{v}(r-r_{0}-tv)\right), & r_{0}\leq r\leq tv+r_{0},\\ 0, & r>tv+r_{0}. \end{array}$$

For some plants with an already established root colonization, the flux of tips at the boundary may even be constant, i.e. *a*=0. In this case, equation (2.10) reduces to

(2.11) $$\rho (r,t)=\frac{vn_{0,b}}{d}\frac{r_{0}}{r}\text{exp}\left(\frac{b}{v}(r-r_{0})\right)\{\begin{array}{cc} 1-\text{exp}\left(\frac{d}{v}(r-r_{0}-tv)\right), & r_{0}\leq r\leq tv+r_{0},\\ 0, & r>tv+r_{0}. \end{array}$$

This solution, expressed only in one-dimensional Cartesian coordinates, has been used by Schnepf & Roose (2006) to develop a sink term for nutrient uptake by a fungal mycelium, which they included in a classical single root nutrient uptake model.

We now consider the general functional form of *f*, which includes nonlinear branching and both types of anastomosis. In this case, the equation for the hyphal tip density is given by

(2.12) $$\frac{\partial n}{\partial t}=-\frac{1}{r}\frac{\partial (vrn)}{\partial r}+b_{n}n\left(1-\frac{n}{n_{\text{max}}}\right)-d_{n}n-a_{2}n\rho -a_{1}n^{2}.$$

Henceforth, we call this model the ‘general model’. Equation (2.12) for the hyphal tip density *n* was solved numerically using a Lax–Wendroff scheme and equation (2.5) for the hyphal length density *ρ* was solved using an explicit Euler scheme (Süli & Mayers 2003). The numerical scheme is presented in appendix B, which was implemented in the programming language Matlab (http://www.mathworks.com).

In this paragraph we discuss the effect of the dimensionless parameters derived in appendices A and B on the model behaviour. We show in appendix A that the growth equations of the linear branching model have only one dimensionless grouping that influences the solution, i.e. *δ*=*d*/*b*. This dimensionless parameter represents the ratio of hyphal death rate to tip branching rate. If *δ*≪1, hyphal death rate is negligible compared with tip branching and biomass is accumulated in the centre of the colony (figure 2*a*). On the other hand, if *δ*≫1, tip branching is negligible compared with hyphal death and biomass is significantly reduced in the centre of the colony and the peak of colony biomass moves away from the root surface. This means that, when *δ* is large, the front of the colony is the most active region even if we assume that hyphae take up nutrients along their entire length. The value of the parameter *δ* is different for different fungal species as we show in §4.

Integrating the solution for the hyphal length density over the colony length, we obtain an equation for the total colony biomass. At any given time, the overall hyphal length *ρ*_*T*_ is given by

(2.13) $$\rho_{T}(t,\delta )=2\pi \int_{r_{0}}^{r_{c}}r\rho (r,t,\delta )\text{d}r,$$

where $r_{c}=vt+r_{0}$ is the edge of the colony. Figure 2*b* shows that the total hyphal length of the colony increases exponentially with time, damped by a factor dependent on the value of the dimensionless parameter *δ*. The smaller the parameter *δ*, the larger the total colony biomass.

We now consider the general model that includes tip–tip and tip–hypha anastomosis. In appendix B, we show that this model is dependent on two additional dimensionless parameters that determine the behaviour of the solution. The parameter $\alpha_{1}=([\rho ]/v)(b_{n}/n_{\text{max}}+a_{1})$ describes the combined effect of nonlinear branching and tip–tip anastomosis in relation to tip growth, and $\alpha_{2}=([\rho ]/b)a_{2}$ describes tip–hypha anastomosis in relation to the linear tip branching rate. Figure 3 shows the effect of varying *α*_1_ on the hyphal length density. If *α*_1_ is small, the result is close to the linear branching model, as expected. The parameter *δ* was set equal to 1, so that the curve in figure 3, where *α*_1_=0, is the same as the curve for *δ*=1 in figure 2*a*. If *α*_1_ is large, the production of new tips is reduced when the tip density is large. Figure 4 shows the effect of varying *α*_2_. When it is small, tip–hypha anastomosis is negligible, and the result is again similar to the linear model. As *α*_2_ increases, the production of new tips and hyphae is increasingly inhibited when both tip and hyphal length densities are large. A typical feature of tip–hypha anastomosis when compared with tip–tip anastomosis is a local peak near the front of the colony.

## 3. Model calibration

AM fungi are obligate symbionts. Therefore, measurement of fungal growth can only be made in the presence of plants and is difficult. Experimental data for calibrating the spatial and temporal explicit models are scarce. Jakobsen *et al.* (1992) present measurements of hyphal length densities of three AM fungi: *Scutellospora calospora* (Nicol. & Gerd.) Walker & Sanders; *Glomus* sp.; and *Acaulospora laevis* Gerdemann & Trappe associated with clover (*Trifolium subterraneum* L.); these data appear suitable for comparison with modelled hyphal length densities. Hyphal length densities were measured at different times and distances from a cylindrical root compartment in a soil that initially had a small mycelium density. Plants with a previously established mycorrhiza were transferred to a two-compartment system, where root growth into a hyphal-only compartment was restricted by a fine nylon mesh. The root compartment was a cylinder with a diameter of 6 cm. The spread of external hyphae from the root compartment into the soil was measured at different distances after 0, 7, 14, 28 and 47 days. The measured values represent the total hyphal length density of mycorrhizal and other soil fungi. A control experiment conducted without mycorrhizae allowed for the estimation of the length densities of mycorrhizal fungi. At 3 cm and 7 days and 2 cm and 14 days, no control values were available. We estimated them by linearly interpolating the neighbouring values to be 1.06 and 2.00 m g^−1^, respectively. The data are presented in units of m g^−1^ dry soil. In order to convert them to units of cm cm^−3^ soil volume as required by the model, we need the soil bulk density (SBD). From the detailed information about the size of the hyphal compartment and amount of soil used, we estimated the SBD to be 1.02 g cm^−3^. Figures 5–7 compare the measured data with model results, which will be explained later in this paper.

For model calibration, we take starting estimates for the model parameters from published data and try to fit both the linear and nonlinear models to measured hyphal length densities. Our initial estimates of *v* come from the adaptation of Jakobsen *et al.* (1992), who measured the spread of hyphal length densities over time. The mean value was 0.08 cm d^−1^ for *S. calospora*, 0.07 cm d^−1^ for *Glomus* sp. and 0.31 cm d^−1^ for *A. laevis*. The guess values for the initial length densities at the root surface were taken to be the measured initial length densities of the three fungal species. Our initial estimate for the death rate *d* is motivated by Staddon *et al.* (2003), who reported that most mycorrhizal hyphae live approximately 6 days. Branching is often measured in number of branches per unit length of hyphae (Giovanetti *et al.* 2001), but we did not find any values for the branching rate. Therefore, we estimate the branching rate by $b_{n}=v/\Delta$, where *v* is the tip elongation rate and *Δ* is the distance between branches. According to Giovannetti *et al.* (2001), estimates for the number of branches per centimetre length of hyphae range from 8.6 to 9.7 for the AM species *Glomus mosseae*. Assuming that they are equally spaced, distances between branches range from 0.115 to 0.132 cm. Using these values and our initial guesses for *v*, our estimates for *b*_*n*_ range from 0.53 to 2.70 d^−1^. To determine an initial estimate for the hyphal tip density initially present at the boundary, we take the measurements of the hyphal length density inside the root compartment to represent the value at the interface between root and fungus compartment. We compute the hyphal tip density from a known value of the hyphal length density as follows. The data show that the hyphal length density at the root–fungus interface increases with time. Following the boundary condition (2.8), we also assume a linear increase in hyphal length density at the boundary with time, i.e.

(3.1) $$\rho (r_{0},t)=\tilde{a}t+\rho_{0,b},$$

where *ρ*_0,*b*_ is the hyphal length density initially present at the root–fungus interface and $\tilde{a}$ is the root proliferation factor for the hyphal length density. This is also justified by the fact that there are only three data points available. However, if required, it would be possible to fit other functions to the data. Inserting equation (3.1) and its time derivative into the model equation for the hyphal length density (2.5), we obtain the following expression for the hyphal tip density at the root–fungus interface:

(3.2) $$n(r_{0},t)=\frac{\tilde{a}d}{v}t+\frac{\tilde{a}+\rho_{0,b}d}{v}.$$

From equation (2.8), we infer that the initial tip density can be calculated from the initial length density as

(3.3) $$n(r_{0},0)=\frac{a}{d}+\frac{\rho_{0,b}d}{v}.$$

We set the initial estimates of the root proliferation parameter *a*, the reciprocal of maximum tip density 1/*n*_max_ and the two anastomosis rates *α*_1_ and *α*_2_ to zero because these parameters represent processes not encountered in all fungal species.

Equation (2.10) as well as the numerical solution of the general model were plotted against the data and the initial estimates of the parameters varied manually until a good fit was obtained. The parameter *v* was fitted first since it is the only parameter that determines the extent of the colony at any given time. Then, the parameters *b*, *d*, *n*_max_, *α*_1_ and *α*_2_ were varied in order to get the correct colony shape. Afterwards, these values were used as initial guesses for the Matlab function ‘fminsearchbnd’ in order to find the parameter set that minimizes the root mean squared error (RMSE) between measured and calculated hyphal length densities. This optimization algorithm uses the Nelder–Mead simplex method (Lagarias *et al.* 1998) and allows that bounds can be applied to the variables. This multistep optimization approach ensured that our numerical optimization algorithm reached a biologically realistic minimum, particularly avoiding negative parameter values. The goodness of fit was assessed using the RMSE,

(3.4) $$\text{RMSE}=\sqrt{\frac{\sum_{i=1}^{n}{\left(\rho_{\text{measured},i}-\rho_{\text{fitted},i}\right)}^{2}}{n}},$$

where *n* is the number of data points.

## 4. Results

We generally found a good agreement between simulated and measured values. The fitted parameter values and the RMSE for the three fungal species are given in table 1.

The linear model represented by equation (2.10) could be fitted to the fungal species *Scutellospora calospora* (figure 5). The RMSE of 1.11 is smaller than the standard error of the mean given for some of the measurements in Jakobsen *et al.* (1992). According to this model, the fungal species *S. calospora* is characterized by linear branching with a relatively small net branching rate *b* when compared with the other species. The dimensionless parameter *δ*=9 is large, which means that hyphal death is large compared with tip branching. The large value of the parameter *a* indicates that growth is mainly supported by a large flux of tips across the root–fungus interface. The fitted value for *v* is approximately three times larger than the value estimated by Jakobsen *et al.* (1992), and the value for *b* that produced a good fit to the measured data is significantly smaller than the initial estimate while the hyphal death rate is close to the initially estimated value.

The linear branching model was not satisfactory for the other two fungal species, *Glomus* sp. and *A. laevis*. When we discuss the general model in the following paragraphs, we consider a combined effect of nonlinear branching and tip–tip anastomosis. In equation (2.12), both tip–tip anastomosis and the nonlinear branching effect depend on *n*^2^. Hence, it is difficult to assign reduction of tips to one or the other effect, and in the following we discuss the two mechanisms as one combined effect as described by the dimensionless parameter $\alpha_{1}=([\rho ]/v)(b_{n}/n_{\text{max}}+a_{1})$. Figure 6 shows the general model fitted to the data of the fungal species *Glomus* sp. In the first few days of the experiment, it still slightly underestimated measured values. In particular, the data point after 7 days at a distance of 1 cm is not reached. However, because the tip density at this point is much larger than any of the other values at later times, we show goodness of fit treating and not treating this data point as an outlier (table 1). When this data point is treated as an outlier, the RMSE of this model is within the range of the standard errors of the mean given for some of the measurements in Jakobsen *et al.* (1992). Following this model, the parameter values in table 1 show that this fungal species is characterized by nonlinear branching/tip–tip anastomosis (the dimensionless parameter *α*_1_=11.52 is large), while no significant tip–hypha anastomosis occurs. The net branching rate *b* is large while fresh supply from the root surface is relatively small. The dimensionless parameter *δ*=0.16 is small, which means that hyphal death is negligibly small. The values for *v* and *d* that produced a good fit to the measured data are approximately two times larger than the initial guess values while the branching rate is within the initially estimated range. Simulated values slightly underestimate measured values in the first few days of the experiment, which indicates that there might be additional relevant processes not yet included in the model.

Figure 7 shows the general model fitted to the data of the fungal species *Acaulospora laevis*. According to the parameter values in table 1, tip–hypha anastomosis is important for this species. We calculate the dimensionless parameter *α*_2_ describing tip–hypha anastomosis in relation to tip branching to be 2.50. The dimensionless parameter *α*_1_ is small, hence nonlinear branching/tip–tip anastomosis is negligible. The dimensionless parameter *δ* is small, hence hyphal death is negligible, and the large value for the parameter *a* indicates that there is a substantial supply of new tips at the root surface. The value for *v* that produced a good fit to the measured data is approximately the same as the initial guess value based on Jakobsen *et al.* (1992). The branching rate also is within the initially estimated range, while the fitted hyphal death rate is half of the initial estimate. A part of the measured behaviour at the later times (28 and 47 days) however could not be captured by the model. The RMSE of this model, 1.33, is equal to the largest standard error of the mean presented for some of the measurements in Jakobsen *et al.* (1992). Again, this indicates that there might be additional relevant processes not yet included in the model or the experimental conditions may have changed during the experiment. However, this will require more substantial changes to the model and to our understanding of how these fungi grow, and we will leave this task for later papers.

## 5. Discussion

We have provided analytical and numerical solutions to a growth model of the external mycelium of AM fungi. We adapted the model of Edelstein (1982) to the case of AM fungi by defining a flux of new hyphal tips at the root–hypha boundary, hence simulating the root surface as a continuous source of new tips. We further extended the model by using time-dependent boundary conditions to make it applicable to the data of Jakobsen *et al.* (1992) for the three fungal species. We found a good agreement between the linear model and the data for *S. calospora*, indicating that nonlinear branching and both kinds of anastomosis are negligible for this fungal species. We also found that it was necessary to adapt the original model equations for the two other fungal species considered. The nonlinear branching/tip–tip anastomosis model could be satisfactorily fitted to the data for *Glomus* sp. and the one including tip–hypha anastomosis performed well for *A. laevis*.

Symbioses between a given host plant and different AM fungi have been shown to differ functionally (Ravnskov & Jakobsen 1995). Such functional differences may be due to the differences between fungi in their relative carbon cost (Munkvold *et al.* 2004), in the rate they colonize roots (Graham & Abbott 2000) and in the production, distribution and function of the external mycelium (Jakobsen *et al.* 1992). This has been confirmed by our model simulations. We showed that the three fungal species considered had different strategies for developing the extraradical mycelium. The parameter values for the species *S. calospora* imply that the fungus is mainly growing and allocating resources for getting a wider catchment area rather than local scavenging, for example, for mineral resources, via branching. Thus, for this fungus, the intraradical mycelium is very important for the function of its extraradical mycelium. Indeed, Jakobsen *et al.* (1992) found that a higher proportion of the root length had become mycorrhizal when compared with the two other fungal species. The parameter values for *Glomus* sp. imply that local scavenging for resources is important for this fungus as long as the hyphal tip density is small. Reaching near the maximum tip density, branching ceases and no more carbon resources are allocated for branching in the respective area. For saprophytic fungi, such local scavenging for resources is important (Donnelly & Boddy 1998). The parameter values for *A. laevis* imply that this fungal species develops a network of interconnected extraradical hyphae via tip–hypha anastomosis. This suggests that nutritional flow between hyphae is important for this fungal species. Note that we consider only anastomosis between hyphae from the same host plant. The formation of anastomosis between AM extraradical mycelium developing from different plants supports the creation of a large interconnected net between plants (Giovanetti *et al.* 2001).

In table 1, fitted rates of tip growth *v* are up to three times larger than the rates of speed measured by Jakobsen *et al.* (1992). However, these measurements are based on the colony-scale behaviour of the mycelium while *v* in the model is a hyphal-scale parameter. The rates of spread estimated by Jakobsen *et al.* (1992) were based on the distance of spread taken to be the distance between the root compartment and the position where the hyphal lengths had declined to half their maximum. This could lead to the underestimation of the rate of hyphal growth. Furthermore, this definition appears to make most sense when the fungal colony declines continuously with distance from the root compartment, which is not the case for all fungal species in these experiments. This highlights the need for care in estimating parameters from experimental data in nonlinear systems and the importance of appropriate mathematical modelling.

Further development of the fungal growth model would consider a more sophisticated description of the extra- and intraradical mycelium. Abbott & Robson (1985) observed significant differences between fungi in the total amount of external mycelium produced as well as in hyphal diameter size classes. This work was extended by Friese & Allen (1991), who described different external hyphal architectures to which they ascribed different functions, such as spread, infection or nutrient absorption. Jakobsen *et al.* (1992) observed significant differences between AM fungi in the spatial distribution of the external mycelium away from roots. This could be linked to differences in phosphate uptake and transport to the plant. All of these studies were conducted with soil-grown plants. With the development of root–organ cultures (ROCs) colonized by AM fungi (Bécard & Piché 1992), the observation and quantification of undisturbed root-external mycelia has been greatly facilitated. This led to detailed descriptions of branched structures that are assumed to play an important role in nutrient absorption (branched absorbing structures (BASs); Bago *et al.* 1998). BASs are an area of our future model development. Recent ROC studies also revealed that AM fungal genera differ greatly in the abundance of hyphal anastomoses (Voets *et al.* 2006). The environment in ROCs is, however, highly artificial and not directly comparable with the soil environment normally encountered by both roots and AM fungi. However, some of the observations done on ROCs regarding differences in extent and interconnectedness of the extraradical mycelium are confirmed in experimental set-ups including soil (Avio *et al.* 2006). The boundary condition, which is now a fitted function, could be replaced by a mechanistic model of root colonization (Neuhauser & Fargione 2004). Furthermore, for applications on the root system scale, it will be necessary to consider that colonization of the root is not homogeneous within the root system and changes over time. However, in the experimental system that we are modelling, the system is thought to be homogeneous along the root and thus this effect can be neglected.

## Figures and Tables

**Figure 1:**
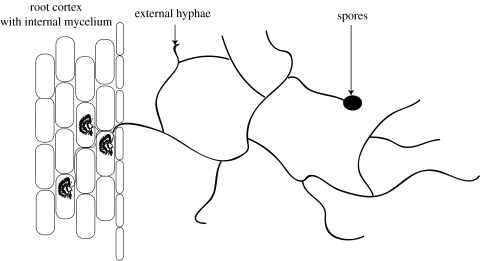
Internal and external mycelium of a vesicular arbuscular mycorrhizal fungus.

**Figure 2:**
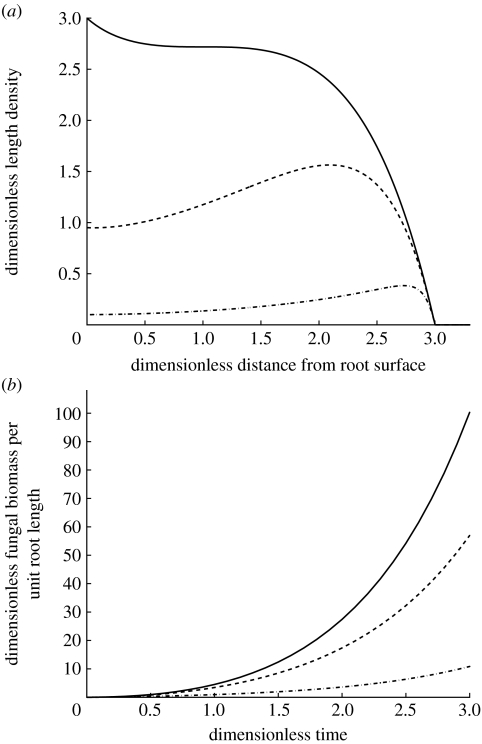
Effects of the dimensionless parameter *δ*. (*a*) Plot of equation (2.11) in dimensionless form. Analytical solution for the hyphal tip and length density in polar radial coordinates for different values of *δ*. Solutions are shown at time *t*=3. (*b*) Overall hyphal length per unit root length for different values of the dimensionless parameter *δ*, computed in equation (2.13). (*a*,*b*) Solid line, *δ*=0.01; dashed line, *δ*=1; dot-dashed line, *δ*=10.

**Figure 3:**
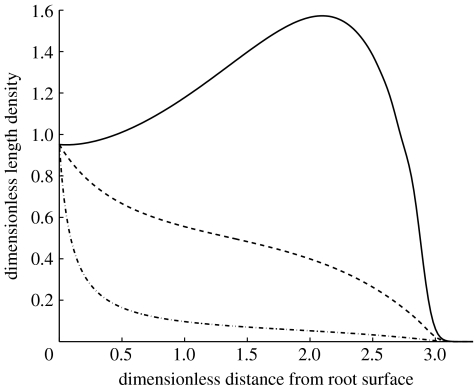
Model including nonlinear branching, tip–tip anastomosis and the effect of varying *α*_1_ (solid line, *α*_1_=0; dashed line, *α*_1_=1; dot-dashed line, *α*_1_=10). Solutions are shown at time *t*=3. Values of other dimensionless parameters are as follows: *δ*=1 and *α*_2_=0. The following are the numerical solution parameters: *J*=300, *μ*=0.8 and Δ*t*=0.01, where *J* is the number of spatial grid points; μ=Δt/Δr; Δ*t* is the time-step size; and Δ*r* is the spatial step size.

**Figure 4:**
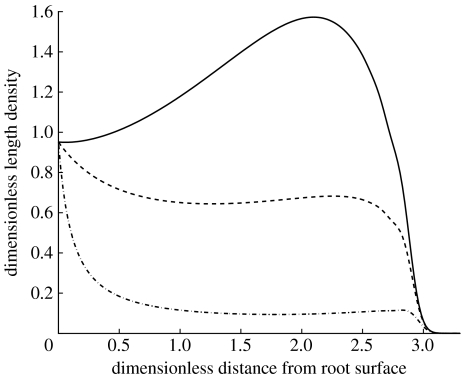
Model including tip–hypha anastomosis and the effect of varying *α*_2_ (solid line, *α*_2_=0; dashed line, *α*_2_=1; dot-dashed line, *α*_2_=10). Solutions are shown at time *t*=3. Values of other dimensionless parameters are as follows: *δ*=1 and *α*_1_=0. Numerical solution parameters are the same as given in figure 3 legend.

**Figure 5:**
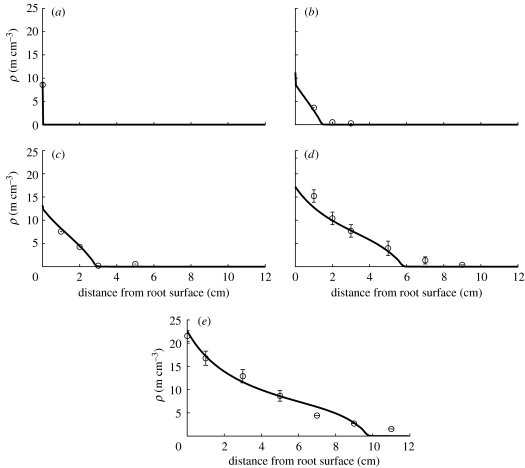
Plot of the linear model given by equation (2.10) in comparison with data for *S. calospora*. See table 1 for corresponding model parameter values. Hyphal length densities at (*a*) 0 days, (*b*) 7 days, (*c*) 14 days, (*d*) 28 days and (*e*) 47 days.

**Figure 6:**
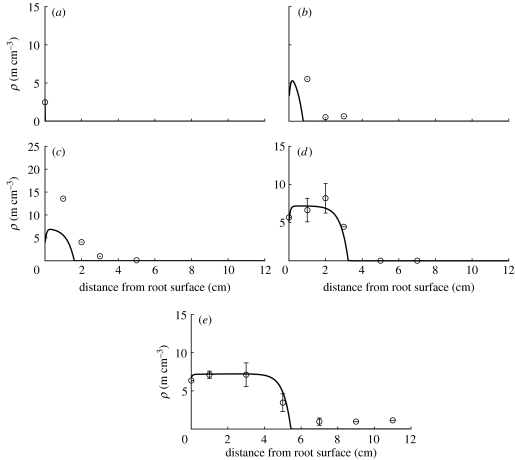
Plot of the general model in comparison with data for *Glomus* sp. See table 1 for corresponding model parameter values. Numerical solution parameters are the same as given in figure 3 legend. Hyphal length densities at (*a*) 0 days, (*b*) 7 days, (*c*) 14 days, (*d*) 28 days and (*e*) 47 days.

**Figure 7:**
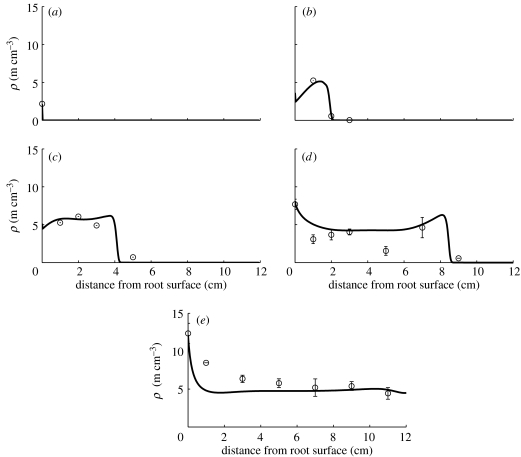
Plot of the general model in comparison with data for *A. laevis*. See table 1 for corresponding model parameter values. Numerical solution parameters are the same as given in figure 3 legend. Hyphal length densities at (*a*) 0 days, (*b*) 7 days, (*c*) 14 days, (*d*) 28 days and (*e*) 47 days.

**Table 1:** Fitted parameter values for the three fungal species considered.

parameter	notation	units	*S. calospora*	*Glomus* sp.	*A. laevis*
speed of tip movement	*v*	cm d^−1^	0.25	0.13	0.28
tip branching rate	*b*_*n*_	d^−1^	0.02	1.91	1.47
tip death rate	*d*_*n*_	d^−1^	5.20×10^−3^	0.15	0.73
net tip branching rate	*b*=*b*_*n*_−*d*_*n*_	d^−1^	0.015	1.76	0.74
hyphal death rate	*d*	d^−1^	0.18	0.28	0.08
maximal hyphal tip density	*n*_max_	cm^−3^	—	2238.14	1.78×10^5^
tip–tip anastomosis rate	*a*_1_	cm^3^ d^−1^	—	—	7.00×10^−7^
tip–hypha anastomosis rate	*a*_2_	cm^3^ m^−1^ d^−1^	—	—	0.15
boundary proliferation	*a*	m cm^−3^ d^−1^	2.52	2.24	0.56
initial tip density at boundary	*n*_0,*b*_	cm^−3^	97.07	84.16	12.68
RMSE			1.11	1.67^a^ 2.38^b^	1.33

a Not including the data point at 1 cm distance after 14 days.

b Including the data point at 1 cm distance after 14 days.

## Appendix A Non-dimensionalization and analytical solution of the linear branching model

We consider equations (2.4)–(2.8) with the functional form of *f* given by *f*=*bn*. If we scale the variables such that

(A1) $$t=\frac{1}{b}t^{*},\;r=\frac{v}{b}r^{*},\;\rho =[\rho ]\rho^{*},\;n=[\rho ]\frac{b}{v}n^{*},$$

where [*ρ*] is a given characteristic hyphal length density, equations (2.4)–(2.8) become (dropping asterisks)

(A2) $$\frac{\partial n}{\partial t}=-\frac{1}{r}\frac{\partial (rn)}{\partial r}+n,\;t\geq 0,\;r\geq {\hat{r}}_{0},$$

(A3) $$\frac{\partial \rho }{\partial t}=n-\delta \rho ,\;t\geq 0,\;r\geq {\hat{r}}_{0},$$

(A4) $$n=0,\;t=0,\;r\geq {\hat{r}}_{0},$$

(A5) $$\rho =0,\;t=0,\;r\geq {\hat{r}}_{0},$$

(A6) $$n=\psi t+\xi ,\;t>0,\;r={\hat{r}}_{0},$$

where δ=d/b, ψ=av/[ρ]b, $\xi =n_{0,b}v/[\rho ]b$ and ${\hat{r}}_{0}=r_{0}b/v$. The parameter δ=d/b describes the relative importance of hyphal death compared with tip branching, *ψ* and *ξ* are dimensionless parameters of the boundary condition and the parameter ${\hat{r}}_{0}$ relates the root radius to the spatial scale.

To solve the hyperbolic equation (A 2), we use the method of characteristics (Ockendon *et al.* 2003). Let *r* and *t* be functions of a parameter *τ*, i.e. *r*=*r*(*τ*), *t*=*t*(*τ*). Then, the characteristic equations are

(A7) $$\frac{\partial t}{\partial \tau }=1,$$

(A8) $$\frac{\partial r}{\partial \tau }=1,$$

and the partial differential equation (A 2) becomes the ordinary differential equation

(A9) $$\frac{\text{d}n}{\text{d}\tau }=\left(1-\frac{1}{r}\right)n.$$

We parametrize the initial condition (A 4) so that at *τ*=0, *r*=*s*, *t*=0 and n(s,0)=0, for ${\hat{r}}_{0}\leq s<\infty$. Integrating the characteristic equations (A 7)–(A 9) gives the parametric solution in the region influenced by the initial condition

(A10) t=τ,

(A11) r=τ+s,

(A12) $$n(s)=0,\;s\geq {\hat{r}}_{0}.$$

We parametrize the boundary condition (A 6) so that at *τ*=0, $r={\hat{r}}_{0}$, *t*=*s* and n(s,0)=ψs+ξ, for $s\leq {\hat{r}}_{0}$. Integrating equations (A 7)–(A 9) gives the parametric solution in the region influenced by the boundary condition

(A13) t=τ+s,

(A14) $$r=\tau +{\hat{r}}_{0},$$

(A15) $$n(s)=\frac{{\hat{r}}_{0}}{r}e^{\tau }(\psi s+\xi ),\;\text{for}\;s\leq {\hat{r}}_{0}.$$

Combining the solutions for the regions influenced by the initial and boundary conditions and eliminating the parameters *τ* and *s*, we have the dimensionless solution for *n*,

(A16) $$n(r,t)=\{\begin{array}{cc} \frac{{\hat{r}}_{0}}{r}e^{\left(r-{\hat{r}}_{0}\right)}(\psi (t-r+{\hat{r}}_{0})+\xi ), & r-t\leq {\hat{r}}_{0},\\ 0, & r-t\geq {\hat{r}}_{0}. \end{array}$$

To find *ρ*, we solve

(A17) $$\frac{\text{d}\rho }{\text{d}t}+\delta \rho =n(r,t),$$

on the interval $\left[r-{\hat{r}}_{0},t\right]$ with initial condition ρ(r,0)=0, for ${\hat{r}}_{0}\leq r\leq \infty$, to obtain the dimensionless solution

(A18) $$\rho (r,t)=\frac{1}{\delta }\frac{{\hat{r}}_{0}}{r}e^{r-{\hat{r}}_{0}}\{\begin{array}{cc} \psi \left(t-r+{\hat{r}}_{0}-\frac{1}{\delta }\right)+\xi +\left(\frac{\psi }{\delta }-\xi \right)e^{\delta (r-{\hat{r}}_{0}-t)}, & r-t\leq {\hat{r}}_{0},\\ 0, & r-t\geq {\hat{r}}_{0}. \end{array}$$

The dimensional solution can be obtained by scaling back to the dimensional variables using the scales given by (A 1).

## Appendix B Numerical solution of the general model

We present a Lax–Wendroff scheme (Morton & Mayers 1994) for the numerical solution of the general model given by equations (2.4)–(2.8) with *f* given by equation (2.12).

Before solving the model, we non-dimensionalize it using the scales given by equation (A 1) and arrive at the following dimensionless equations:

(B1) $$\frac{\partial n}{\partial t}=-\frac{1}{r}\frac{\partial (rn)}{\partial r}+n-\alpha_{1}n^{2}-\alpha_{2}n\rho ,\;t\geq 0,\;r\geq {\hat{r}}_{0},$$

(B2) $$\frac{\partial \rho }{\partial t}=n-\delta \rho ,\;t\geq 0,\;r\geq {\hat{r}}_{0},$$

(B3) $$n=0,\;t=0,\;r\geq {\hat{r}}_{0},$$

(B4) $$\rho =0,\;t=0,\;r\geq {\hat{r}}_{0},$$

(B5) $$n=\psi t+\xi ,\;r={\hat{r}}_{0},\;t>0,$$

where $\alpha_{1}=([\rho ]/v)(b_{n}/n_{\text{max}}+a_{1})$ is the combined effect of nonlinear branching and tip–tip anastomosis in relation to tip movement and $\alpha_{2}=a_{2}([\rho ]/b)$ describes tip–hypha anastomosis in relation to tip branching.

Let *T*_f_ be the final time, *M* the number of time steps, Δ*r* the grid spacing, *J* the number of grid points and $\Delta t=T_{\text{f}}/M$. Let $n(r_{j},t_{m})\approx U_{j}^{m}$, $\rho (j_{j},t_{m})\approx V_{j}^{m}$ and μ=Δt/Δr. The scheme for *U* is based on substituting for the time derivatives

(B6) $$U_{t}=-\frac{1}{r}\frac{\partial }{\partial r}(rU)+U-\alpha_{1}U^{2}-\alpha_{2}UV,$$

(B7) $$U_{tt}=-\frac{1}{r}\frac{\partial }{\partial r}(rU_{t})+U_{t}-2\alpha_{1}UU_{t}-\alpha_{2}U_{t}V-\alpha_{2}UV_{t},$$

where $V_{t}=U-\delta V$, into the Taylor series expansion $U_{j}^{m+1}=U_{j}^{m}+\Delta t{\left[U_{t}\right]}_{j}^{m}+({\left(\Delta t\right)}^{2}/2){\left[U_{tt}\right]}_{j}^{m}+O({\left(\Delta t\right)}^{3})$. Neglecting higher-order terms, replacing the space derivatives by centred differences and rearranging gives the Lax–Wendroff scheme

$$U_{j}^{m+1}=\left(1+\Delta t-\mu^{2}+\frac{{\left(\Delta t\right)}^{2}}{2}\right)U_{j}^{m}+\left(-\Delta t\frac{\mu }{2}-\frac{\mu }{2}+\frac{\mu^{2}}{2}\right)\frac{r_{j+1}^{m}}{r_{j}^{m}}U_{j+1}^{m}+\left(\Delta t\frac{\mu }{2}+\frac{\mu }{2}+\frac{\mu^{2}}{2}\right)\frac{r_{j-1}^{m}}{r_{j}^{m}}U_{j-1}^{m}+\left[-\Delta t\alpha_{1}-\frac{3}{2}{\left(\Delta t\right)}^{2}\alpha_{1}-\frac{{\left(\Delta t\right)}^{2}}{2}\alpha_{2}\right]{\left(U_{j}^{m}\right)}^{2}+\left[-\Delta t\alpha_{2}-{\left(\Delta t\right)}^{2}\alpha_{2}+\frac{{\left(\Delta t\right)}^{2}}{2}\alpha_{2}\delta \right]U_{j}^{m}V_{j}^{m}+\left[\frac{1}{4}\Delta t\mu \alpha_{1}\right]\frac{r_{j+1}^{m}}{r_{j}^{m}}{\left(U_{j+1}^{m}\right)}^{2}-\left[\frac{1}{4}\Delta t\mu \alpha_{1}\right]\frac{r_{j-1}^{m}}{r_{j}^{m}}{\left(U_{j-1}^{m}\right)}^{2}+\left[\frac{1}{4}\Delta t\mu \alpha_{2}\right]\frac{r_{j+1}^{m}}{r_{j}^{m}}U_{j+1}^{m}V_{j+1}^{m}-\left[\frac{1}{4}\Delta t\mu \alpha_{2}\right]\frac{r_{j-1}^{m}}{r_{j}^{m}}U_{j-1}^{m}V_{j-1}^{m}+\left[\frac{1}{2}\Delta t\mu \alpha_{1}\right]\frac{r_{j+1}^{m}}{r_{j}^{m}}U_{j+1}^{m}U_{j}^{m}-\left[\frac{1}{2}\Delta t\mu \alpha_{1}\right]\frac{r_{j-1}^{m}}{r_{j}^{m}}U_{j-1}^{m}U_{j}^{m}+\left[\frac{1}{4}\Delta t\mu \alpha_{2}\right]\frac{r_{j+1}^{m}}{r_{j}^{m}}U_{j+1}^{m}V_{j}^{m}-\left[\frac{1}{4}\Delta t\mu \alpha_{2}\right]\frac{r_{j-1}^{m}}{r_{j}^{m}}U_{j-1}^{m}V_{j}^{m}+{\left(\Delta t\right)}^{2}\alpha_{1}^{2}{\left(U_{j}^{m}\right)}^{3}+\frac{3}{2}{\left(\Delta t\right)}^{2}\alpha_{1}\alpha_{2}{\left(U_{j}^{m}\right)}^{2}V_{j}^{m}+\frac{{\left(\Delta t\right)}^{2}}{2}\alpha_{2}^{2}{\left(V_{j}^{m}\right)}^{2}U_{j}^{m}.$$
